# Pathogenic Mechanism and Multi-omics Analysis of Oral Manifestations in COVID-19

**DOI:** 10.3389/fimmu.2022.879792

**Published:** 2022-07-04

**Authors:** Ming Hao, Dongxu Wang, Qianyun Xia, Shaoning Kan, Lu Chang, Huimin Liu, Zhijing Yang, Weiwei Liu

**Affiliations:** ^1^ Department of Oral and Maxillofacial Surgery, Hospital of Stomatology, Jilin University, Changchun, China; ^2^ Laboratory Animal Center, College of Animal Science, Jilin University, Changchun, China; ^3^ Jilin Provincial Key Laboratory of Tooth Development and Bone Remodeling, Hospital of Stomatology, Jilin University, Changchun, China

**Keywords:** COVID-19, SARS-CoV-2, immune response, multi-omics, inflammation

## Abstract

Coronavirus disease 2019 (COVID-19) is a respiratory infectious disease that seriously threatens human life. The clinical manifestations of severe COVID-19 include acute respiratory distress syndrome and multiple organ failure. Severe acute respiratory syndrome coronavirus 2 (SARS-CoV-2), the causal agent of COVID-19, spreads through contaminated droplets. SARS-CoV-2 particles have been detected in the saliva of COVID-19 patients, implying that the virus can infect and damage the oral cavity. The oral manifestations of COVID-19 include xerostomia and gustatory dysfunction. Numerous studies showed that the four structural proteins of SARS-CoV-2 are its potential pathogenic factors, especially the S protein, which binds to human ACE2 receptors facilitating the entry of the virus into the host cells. Usually, upon entry into the host cell, a pathogen triggers the host’s immune response. However, a mount of multi-omics and immunological analyses revealed that COVID-19 is caused by immune dysregulation. A decrease in the number and phenotypes of immune cells, IFN-1 production and excessive release of certain cytokines have also been reported. In conclusion, this review summarizes the oral manifestations of COVID-19 and multi-omics analysis of SARS-CoV-2 infection.

## 1 Introduction

Coronavirus disease 2019 (COVID-19) is an ongoing pandemic caused by the severe acute respiratory syndrome coronavirus 2 (SARS-CoV-2) (1, 2). SARS-CoV-2 is a kind of zoonotic virus affecting both humans and animals (3). It mainly infects the respiratory tract (4), the nervous system (5, 6), and the gastrointestinal tract (7). COVID-19 can develop into acute respiratory distress syndrome (ARDS), causing multiple organ failure and death (8). Since the oral cavity is directly connected to the external environment, it is easy to come into contact with viruses and other microorganisms through the oral cavity, including herpesvirus, retrovirus, cytomegalovirus, influenza virus, etc. (9). A variety of viruses can infect oral mucosa and salivary glands, causing oral symptoms. SARS-CoV-2 can be transmitted through droplets, aerosols, and contact with contaminated surfaces. Therefore, growing evidence suggests that the SARS-CoV-2 infection occurs when a person touches surfaces contaminated with SARS-CoV-2 and then directly touches the mucous membranes of the oral cavity and nose (10, 11). In addition to affecting the respiratory and immune systems, COVID-19 is manifested through different oral pathological features, including gustatory dysfunction, xerostomia, and salivary gland diseases (9, 12).

SARS-CoV-2 is a member of β-coronavirus genus (13). It contains four major structural proteins, including the spike (S) protein (14), which is an important virulence factor of SARS-CoV-2, mediating the entry of the virus into the host cells (4). Increasing evidence suggests that the occurrence and development of COVID-19 are related to the immune dysregulation caused by SARS-CoV-2 (15, 16). SARS-CoV-2 inhibits the secretion of type I interferon (IFN-1) and causes the cytokine storm (17, 18). Since the binding of SARS-CoV-2 to the oral cavity host cells is mediated by the angiotensin-converting enzyme 2 (ACE2) receptors (19), the virus can infect the epithelial cells of the oral mucosa and salivary glands, especially the epithelial cells of the tongue (20–22). In this review, we summarize the oral manifestations of COVID-19 and clarify the etiology and immunological pathogenesis of COVID-19 using multi-omics analysis.

## 2 Oral Manifestations of COVID-19

COVID-19 is a respiratory disease that manifests with fever, cough, dyspnea, headache, chest discomfort, and general body pain (23). Loss of taste and smell in early COVID-19 infection has been reported in some patients (24). A systematic analysis of COVID-19 clinical symptoms revealed that some patients present with unique symptoms, including oral disorders, such as gustatory dysfunction, oral mucosal diseases, salivary gland diseases, gingivitis, and periodontitis (9, 25).

### 2.1 Gustatory Dysfunction

Gustatory dysfunction is one of the most common oral manifestations of COVID-19 (26). Some COVID-19 patients reported taste and smell dysfunctions (25, 27–29). Given the increase in the number of COVID-19 patients with taste and smell dysfunctions, the Centers for Disease Control and Prevention (CDC) has included “New loss of taste or smell” as a symptom of COVID-19 diagnosed as SARS-CoV-2 infection. In one research involving 69 patients with olfactory and taste dysfunctions, 75.4% were diagnosed with COVID-19 (30). In addition, gustatory dysfunction can be used as a criterion for diagnosing COVID-19 (31). Overall, these findings suggested that gustatory dysfunction is a critical symptom of COVID-19, which may be helpful for the diagnosis of COVID-19.

### 2.2 Salivary Gland Diseases

Xerostomia is a common oral symptom of the early stage of COVID-19 disease (22, 25, 32, 33). A report showed the appearance of xerostomia symptoms in COVID-19 (34). In one research, over 70% of patients with xerostomia and loss of taste and smell tested positive before the COVID-19 diagnosis (35). Therefore, xerostomia and taste and smell dysfunctions are prodromal or unique early symptoms of COVID-19 and can be relied on to control the spread of the virus.

Dysphagia and frequent swelling or pain in the salivary glands or face are other oral COVID-19-related symptoms (36, 37). Salivary gland ectasia is a common oral manifestation (32). Reports of COVID-19-related parotitis and sialadenitis of the submandibular gland suggest that acute parotitis may be an early manifestation of COVID-19 (38, 39). In an analysis of oral involvement, salivary gland ectasia was observed in 43% of COVID-19 patients, suggesting that excessive inflammatory response in the salivary glands may indicate SARS-CoV-2 (32). Interestingly, SARS-CoV-2 virions have been detected in the patients’ saliva prior to the apparent lung lesions, which may be caused by SARS-CoV-2 infection in the salivary glands, explaining the asymptomatic COVID-19 infection (40). These reports show that oral diseases may be directly related to SARS-CoV-2 infection. These findings suggest that certain oral symptoms are strong indicators of SARS-CoV-2 infection. SARS-CoV-2 enters the host cells *via* ACE2 receptors abundant in the epithelial cells of the oral cavity, which might explain the involvement of the oral cavity in SARS-CoV-2 infection.

## 3 Structure of SARS-CoV-2

**Figure 1:**
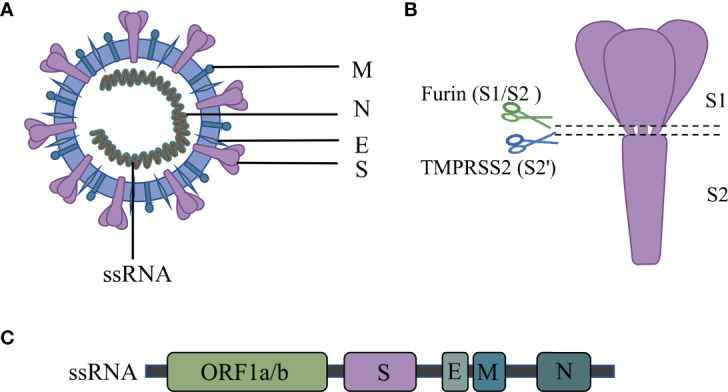
Structure of the SARS-CoV-2.

The SARS-CoV-2 is a single-stranded RNA virus. Its RNA encodes four major structural proteins, which include spike protein (S), envelope protein (E), membrane protein (M), and nucleocapsid protein (N) (41) [**Figure 1**]. Besides, 16 nonstructural proteins (NSPs) and 9 accessory proteins are included in the 29 proteins encoded (42).

The S protein mediates the virus’s entry into host cells and plays a key role in coronavirus infection (43). The S protein comprises the S1 receptor binding subunit and the S2 membrane fusion subunit (44). SARS-CoV-2 binds to the ACE2 receptor *via* the RBD region on the S1 subunit (45). The S2 subunit fuses with the host and viral membranes, facilitating the delivery of the viral genome into the host cells (43). The S protein is thus a vital component of the SARS-CoV-2 virus pathogenicity and might be used for COVID-19 diagnosis.

The E protein participates in the infection, replication, assembly, release, and virulence effect of the SARS-CoV-2 life cycle (46, 47). The E protein mediates the assembly and budding of the virus by interacting with the M protein (48). Moreover, E protein induces the host immune responses by promoting the activation of the NLRP3 inflammasome (49–51). Inhibiting or loss of expression of the E protein reduces titers of virions and induces incomplete viral maturation (52, 53).

Like the E protein, the M protein also inhibits the innate immune response. For instance, the M protein suppresses the signal transduction of RIG-I and MDA5 by targeting the mitochondrial antiviral signaling (MAVS) protein and then inhibits the virus-induced activation of the IFN-β promoter (54, 55).

The N protein has two main functions: it mediates the assembly of the helical capsid around the viral RNA and regulates the transcription of the viral genome (56). Also, the N protein promotes the expression of cytokines by activating the NLRP3 inflammasome signaling pathway (57). The nucleocapsid (N) proteins have dual regulatory effects on the innate immune response. At a low dose, the N protein inhibits the expression of IFN-1; however, at a high concentration, the N protein promotes the secretion of IFN-1 and cytokine release (58).

In addition to structural proteins, NSPs and accessory proteins of SARS-CoV-2 have a role in pathogenicity by influencing the host cell signaling (59). In general, the SARS-CoV-2 proteins play different critical roles in the immune invasion of the virus and modulation of the host immune response. Therefore, understating the role of SARS-CoV-2 proteins can lead to the identification of important diagnostic and therapeutic targets for vaccines against COVID-19.

For example, subunit vaccines, viral vector vaccines and inactivated viral vector vaccines induce antibodies targeting the S protein of SARS-CoV-2 (60–66).

## 4 Multi-omics Analysis of COVID-19

Multi-omics analysis reveals the pathogenic mechanism of organisms, including how they evade the immune system.

Transcriptomics, proteomics, metabolomics, immunomics, and single-cell transcriptomics are useful tools for analyzing biomolecules such as mRNAs, proteins, metabolites, and single cells (67) (**Table 1**). Therefore, they can clarify the pathogenesis and progression of COVID-19. Bronchoalveolar lavage fluid (BALF) and peripheral blood mononuclear cells (PBMC) of COVID-19 patients are common samples used for analyses (68, 74).

**Table 1:** Summary of the main multiple omics data about COVID-19.

Omics Application	Biospecimen Types	Reference
multi-omics (proteomics, metabolomics, single-cell RNA-seq, single-cell TCR-seq, single-cell secretome)	plasma, PBMC	(68)
multi-omics (transcriptomics, proteomics, metabolomics, lipidomics)	blood	(69)
multi-omics (metabolomics, proteomics, lipidomics)	red blood cells	(70)
multi-omics (metabolomics, lipidomics)	serum	(71)
multi-omics (metabolomics, proteomics)	serum, urine	(72)
transcriptomic	whole blood cell, granulocyte preparations	(73)
single-cell RNA-seq (scRNA-seq)	nasal, bronchoalveolar lavage fluid (BALF), PBMCs	(74)
selective spatial transcriptomic	lung biopsies	(75)
shotgun transcriptome, spatial omics	clinical specimens, autopsy tissues	(76)
comparative genomics	SARS-CoV-2 viruses	(77)

### 4.1 The Target Cells Infected by SARS-CoV-2 in Oral Cavity

A study on Rhesus Macaques demonstrated that ACE2 (+) epithelial cells in salivary glands duct were the early target cells of SARS-CoV infection (78). SARS-CoV-2 is also recognized by ACE2 receptors. These findings suggest that SARS-CoV-2 targets ACE2 (+) salivary glands duct epithelial cells. Single-cell RNA sequencing (scRNA-seq) was used to evaluate the specific expression of ACE2 in oral cells. The data showed that compared to buccal and gingiva tissues, the expression of ACE2 was higher in tongue tissues (20). Interestingly, analysis of 7 kinds of cell lines of oral cavity showed that the expression of ACE2 was enriched in epithelial cells (20). This finding indicates that SARS-CoV-2 has ability to influence oral epithelial cells which is a potential pathway of SARS-CoV-2 infection in oral cavity. Evidence suggested that Furin could promote the virus-cell fusion by acting on the cleavage site of S protein to make the virus enter the target cell (79). ScRNA-seq and immunohistochemical (IHC) analysis of oral cells showed that ACE2 receptors, Furin and TMPRSS2 were enriched in oral mucosal and salivary glands cells, especially in epithelial cells (80, 81). Therefore, these data indicate that ACE2 receptor, Furin and TMPRSS2 play an essential role in SARS-CoV-2 infection in oral epithelial cells. In addition, a report showed S protein of SARS-CoV-2 had been detected in epithelial cells of dorsum of the tongue (82). Moreover, a previous study showed that SARS-CoV-2 could infect epithelial cells *in situ* and then shed into saliva which confirmed by scRNA-seq, orthogonal RNA, and protein expression analysis (83). Furthermore, it was demonstrated the inhibited expression of ACE2 and Furin through Maackia amurensis seed lectin (MASL) which has a potential therapeutic effect on COVID-19 by decreasing the expression of inflammatory mediators by oral epithelial cells (84). Considering of host response in SARS-CoV-2 infection, scRNA-seq and transcriptomic analysis were performed. The data showed that upregulated pro-inflammatory signaling and immune dysregulation were observed in epithelial cells of the lung (85, 86). Moreover, the expression of proinflammatory cytokine genes was demonstrated in gingival epithelial cells, which also confirmed the antiviral defense mechanism in oral cavity (87). Besides, nCounter analysis of oral mucosa in severe patients showed signals of cell arresting which was correlated with systemic immune response abnormalities (88). Furthermore, the intense lymphocytic infiltration was detected in minor salivary glands (89). These studies indicate that SARS-CoV-2 could infect oral epithelial cells and be involved in abnormal immune regulation.

### 4.2 Omics Analysis of the Immune Response in COVID-19

Proteomic analysis of COVID-19 patients has shown that high levels of viremia are associated with sustained elevated levels of certain entry factors, such as ACE2 receptor, Furin and cathepsin B/L (CTSB/CTSL) (90). Previous report demonstrated that SARS-CoV-2 failed to enter cells which loss expressed ACE2 receptor (91). These results suggested that ACE2 receptor of host cell has a role in the infection of SARS-CoV-2. In addition, research shows that IFN-1 and IFN-III are under-expressed, whereas inflammatory cytokines such as IL-6, IL1RA, CCL2, CCL8, CXCL2, CXCL8, CXCL9, and CXCL16 are overexpressed in the serum of COVID-19 patients (17, 92). Furthermore, CCL4, CXCL10, IL-7, and IL-1α exacerbate the COVID-19 disease (93).

A positive correlation has been reported between the proliferation of monocytes and DCs that express MKI67 and TOP2A and the severity of COVID-19 disease (93). A decrease in the proportion of CD21^+^ and CD27^+^ B cells has been reported in the moderate and severe COVID-19 cases (94). Compared with moderate and mild COVID-19, the expansion of plasmablasts and plasma cells is lower than that in critical and severe cases (93). A similar trend is observed for B cell response to IFN-α (93).

Compared with healthy or patients with mild COVID-19, there is a decrease in the proportion of T lymphocytes, monocytes, dendritic cells, and natural killer cells, but a significant increase in neutrophils, hyperactivated T cells, and cytotoxic CD8^+^ T cells in patients with severe COVID-19 (94, 95). The proportion of lymphocytes also changes in COVID-19 patients, which shows that the proportion of CD4-CTLs increased, whereas the proportion of reactive Treg cells decreased (96). T-cell signaling is present in mild patients, but absent in severe patients (97). Moreover, both NLR (neutrophil count-to-lymphocyte count ratio) and NTR (neutrophil-to-T cell ratio) are elevated in severe COVID-19 patients (94). Neutrophilia and lymphocyte dysfunction may be related to tissue damage caused by the massive release of cytokines. High plasmablasts, circulating megakaryocytes, and erythropoiesis have been reported in severe COVID-19 cases (17, 69, 97–99).

In fact, the progression of the SARS-CoV-2 infection differs among patients. Multi-omics can reveal the changes in the increased secretion of cytokines, an increased proportion of neutrophils, and a decreased proportion of lymphocytes, which can open up new horizons in treating COVID-19 and the pathogenic mechanism of SARS-CoV-2. In the present study, the multi-omics analysis revealed increased secretion of cytokines and the decreased expression of IFN, respectively, in COVID-19 patients, further indicating that SARS-CoV-2 affects the function of the immune system.

### 4.3 Omics Analysis of Biomarkers of COVID-19

Notably, the potential therapeutic and diagnostic markers of COVID-19 were screened by omics (**Table 2**). Considering the invasion of SARS-CoV-2 in mammalian cells, omics analysis is a powerful tool for studying the roles of ACE2 receptor, cathepsin L1 (CTSL), and transmembrane serine proteinase 2 (TMPRSS2) (74). Furthermore, proteomic analysis of COVID-19 patients revealed a significant increase in cathepsin L1 in the lung (109). Thus, ACE2 receptors, CTSL 1, and TMPRSS2 can be targets for preventing and treating COVID-19. Moreover, studies have shown that soluble ACE2 and TMPRSS2 inhibitors have antiviral effects by blocking viral infection (19, 110). Proteomic analysis of SARS-CoV-2-infected host cells revealed that SARS-CoV-2 reshapes central cellular pathways of translation, splicing, carbon metabolism, protein homeostasis, and nucleic acid metabolism (100). In addition, the application of translation inhibitors significantly inhibits the replication of SARS-COV-2 (100). Multi-omics analysis of SARS-COV-2-infected cells showed that CIGB-300 interferes with RNA splicing by targeting casein kinase II (CK2) at the early stage of viral infection, suggesting that cigB-300 has antiviral effects (101). Transcriptome analysis showed increased HSP90AA1 mRNA levels in virus-infected cells, reducing viral replication and pro-inflammatory cytokine expression by inhibiting HSP90 activity (102).

**Table 2:** Summary of the main multiple omics data about biomarkers of COVID-19.

Omics	Biomarkers	Application	Reference
transcriptomics	ACE2 receptor	therapy	(20)
scRNA-seq	ACE2 receptor, TMPRSS	therapy	(83)
proteomics	translation, splicing	therapy	(100)
multi-omics	CK2	therapy	(101)
transcriptomics	HSP90	therapy	(102)
multi-omics (interactome, phosphoproteome, ubiquitylome, transcriptome, and proteome)	NSP3	therapy	(103)
structural genomics	ORF9b, Nsp1, Nsp7, Nsp8, Nsp12, S protein	therapy	(104)
proteomics	Tenascin-C (TNC), Mucin-1(KL-6)	therapy	(105)
proteomics	peptides from SARS-CoV-2 nucleoprotein	diagnosis	(106)
proteomics, transcriptomics	S100s	diagnosis	(107)
Ultra-High-Throughput proteomics	ALB, APOA1, APOC1, GSN, TF	diagnosis	(108)

In addition, multi-omics can be used to reveal the progression of COVID-19. For example, the mRNA level of S100s (107), pro-inflammatory signaling molecules of IL-6 are upregulated, and down-regulation of proteins in albumin (ALB), apolipoprotein A1 (APOA1), apolipoprotein C1 (APOC1), gelatins (GSN) and transferrin (TF) is seen in severe COVID-19 disease (108). These biomarkers have potential applications in the diagnosis of COVID-19.

## 5 Pathogenic Mechanisms in COVID-19

SARS-CoV-2 infection induces several immune responses. Firstly, upon entry into the body, the antigen-presenting cells (APCs) recognize the pathogen-associated molecular patterns (PAMPs) of SARS-CoV-2 through multiple pattern recognition receptors (PRRs) (111). Activated immune cells then produce numerous cytokines, such as IFNs, TNF-α, and interleukins, to destroy the virus- infected cells (112–114). The pathogenesis of SARS-CoV-2 is related to the inhibition of IFN production and the related cytokine storm (115).

### 5.1 SARS-CoV-2 Receptors

**Figure 2:**
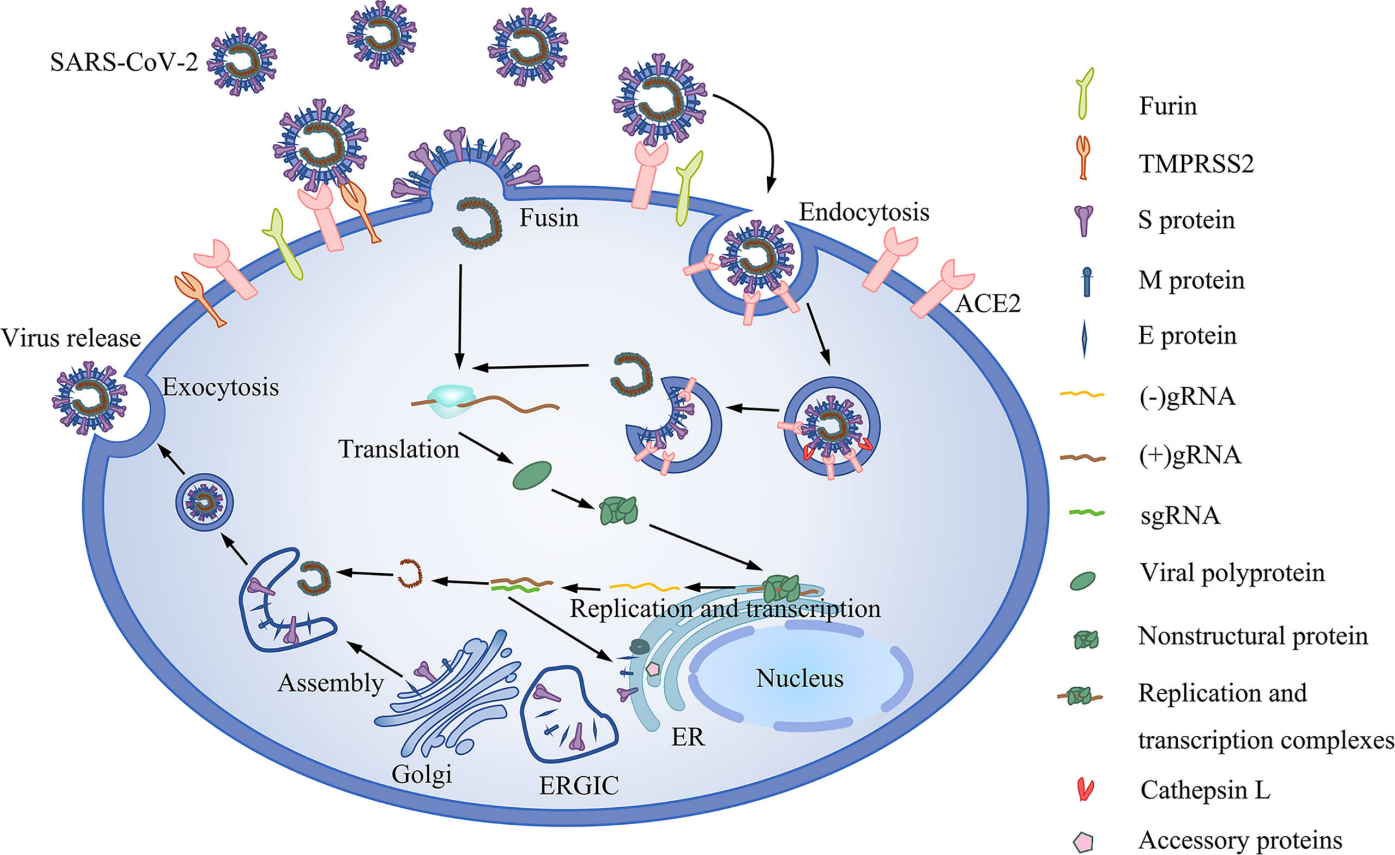
The life cycle of SARS-CoV-2. It includes viral entry, replication and transcription, assembly and release. Binding of SARS-CoV-2 to the ACE2 receptor and the subsequent activation of the virus by Furin and TMPRSS2. In the absence of TMPRSS2, the virus is activated by intracellular cathepsin. Upon entry into the cell, ORF1ab of the virus is translated to polyproteins, which are then cleaved into nonstructural proteins before assembly into replication and transcription complexes. Replication and transcription of the genome generate gRNA and subgenomic RNA (sgRNA). Shorter sgRNAs encode structural proteins and accessory proteins. The ERGIC is then assembled into mature SARS-CoV-2 virions.

Studies have shown that the ACE2 receptor is the cellular receptor of SARS-CoV-2 (116, 117). ACE2 is expressed on the oral mucosa and salivary gland cells, suggesting that the oral cavity participates in the SARS-CoV-2 infection (20, 21). Once in the body, S protein is activated by TMPRSS2, which promotes the release of the SARS-CoV-2 genome into host cells (118) [**Figure 2**]. In general, ACE2 and TMPRSS2 are critical for SARS-CoV-2 infection. Reports show that ACE2 and TMPRSS2 are both expressed on the epithelial cells of the oral mucosa and salivary glands (83, 119, 120). ACE2 and TMPRSS2 are both expressed in taste buds cells; moreover, ACE2 is highly enriched in the epithelial cells of the tongue, which may be related to gustatory dysfunction (121). Interestingly, the expression of ACE2 on small salivary glands is higher than that in lungs, and the positive rate of SARS-CoV-2 in the saliva of asymptomatic infected patients is as high as 91.7% (40). The above findings underscore the critical role of ACE2 receptors in SARS-CoV-2.

### 5.2 Analysis of Pathological Process in SARS-COV-2 Infected Oral Cells

After SARS-CoV-2 infects oral cells by recognizing of ACE2 receptors, it causes damage to tissues or cells, thus leading to oral manifestations of COVID-19. ACE2 specific antibody test proved that the gustatory dysfunction of COVID-19 patients was related to the directly infected human taste cells in the dorsum of the tongue (122). Moreover, SARS-CoV-2 was also detected in submandibular gland of the COVID-19 patients (123, 124). Besides, IHC analysis of lip tissues with blister-like lesions showed that SARS-CoV-2 spike protein was positive in minor salivary acinus and duct cells (125). Interestingly, micronucleus test demonstrated that the death of oral mucosal cells was induced by SARS-CoV-2 (126). These indicate that SARS-CoV-2 induces cell death when it infects the salivary glands. Moreover, it was proposed that the infected salivary gland epithelial cells lysis stimulated the excessive secretion of inflammatory cytokines, causing salivary gland tissue damage (127). More importantly, *in situ* hybridization (ISH) and immunophenotyping showed that the most common histological feature of infected salivary glands was chronic salivary gland inflammation including lymphocytic inflammation and epithelial injury (83). These indicate that SARS-CoV-2 infection results in salivary gland dysfunction and xerostomia through excessive inflammatory response and the direct damage to ducts and acinar cells.

### 5.3 The Innate Immune Response Induced by SARS-CoV-2

IFN-1 is an important component of the innate immune response against viral infections. Recognition of PAMPs *via* the PRRs rapidly triggers the release of IFN-1 and many other pro-inflammatory cytokines, including interleukin (IL)-1β, IL-2, IL-6, IL-7, granulocyte colony-stimulating factor (GCSF), IFN-γ, and tumor necrosis factor-α (TNF-α) (128, 129). PRRs include Toll-like receptors (TLR), retinoic acid-inducible gene I (RIG-I)-like receptors (RLR), and C-type lectin receptors (CLR) (130, 131). IFN can regulate antiviral T cell responses and induce the expression of interferon-stimulated genes (ISG) *via* the JAK/STAT signaling pathway (132–134).

**Figure 3:**
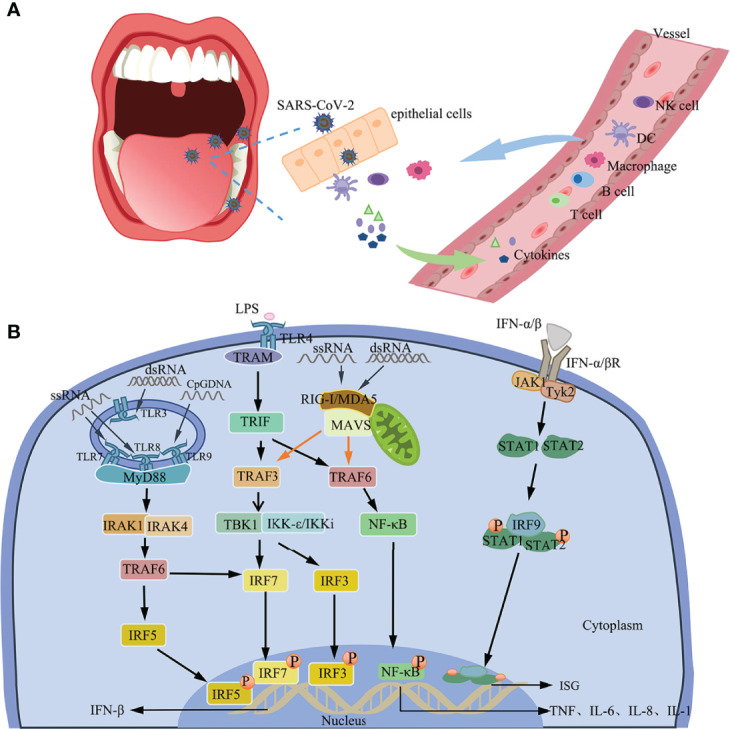
Immune response. **(A)** Immune response to SARS-CoV-2 in the oral cavity. **(B)** IFN induction and the positive feedback signaling pathway. The production of IFN-β by TLR4-TBK1/IKKi, TLR7/8/9-MyD88/IRAK1/IRAK4, and RIG-I/MDA5/MAVS signals. IFN-1 induces the expression of ISG *via* the Tyk2/JAK1/STAT signaling pathway by binding to IFNARs.

TLRs recruit specific adaptor molecules of downstream of the signaling cascade to initiate innate immune responses *via* the TLR/MyD88/NF-κB and TRIF/IFN-β pathway signaling pathways (135) [**Figure 3**]. Apart from TLRs, immune cells often recognize PAMPs *via* the RLRs, which induces the production of IFN. The RIG-I and MDA5 are TLRs that recognize and initiate an immune response against SARS-CoV-2 (136). Activated RIG-I and MDA5 interact with the downstream adapter MAVS to induce the expression of IFN-β and early ISGs (134) [**Figure 3**].

### 5.4 Adaptive Immunity Against SARS-CoV-2

Innate immunity performs two main functions: it directly kills pathogens and initiates adaptive immune responses (137). Adaptive immunity comprises humoral immunity and cellular immunity.

#### 5.4.1 Cellular Immunity Against SARS-CoV-2

**Figure 4:**
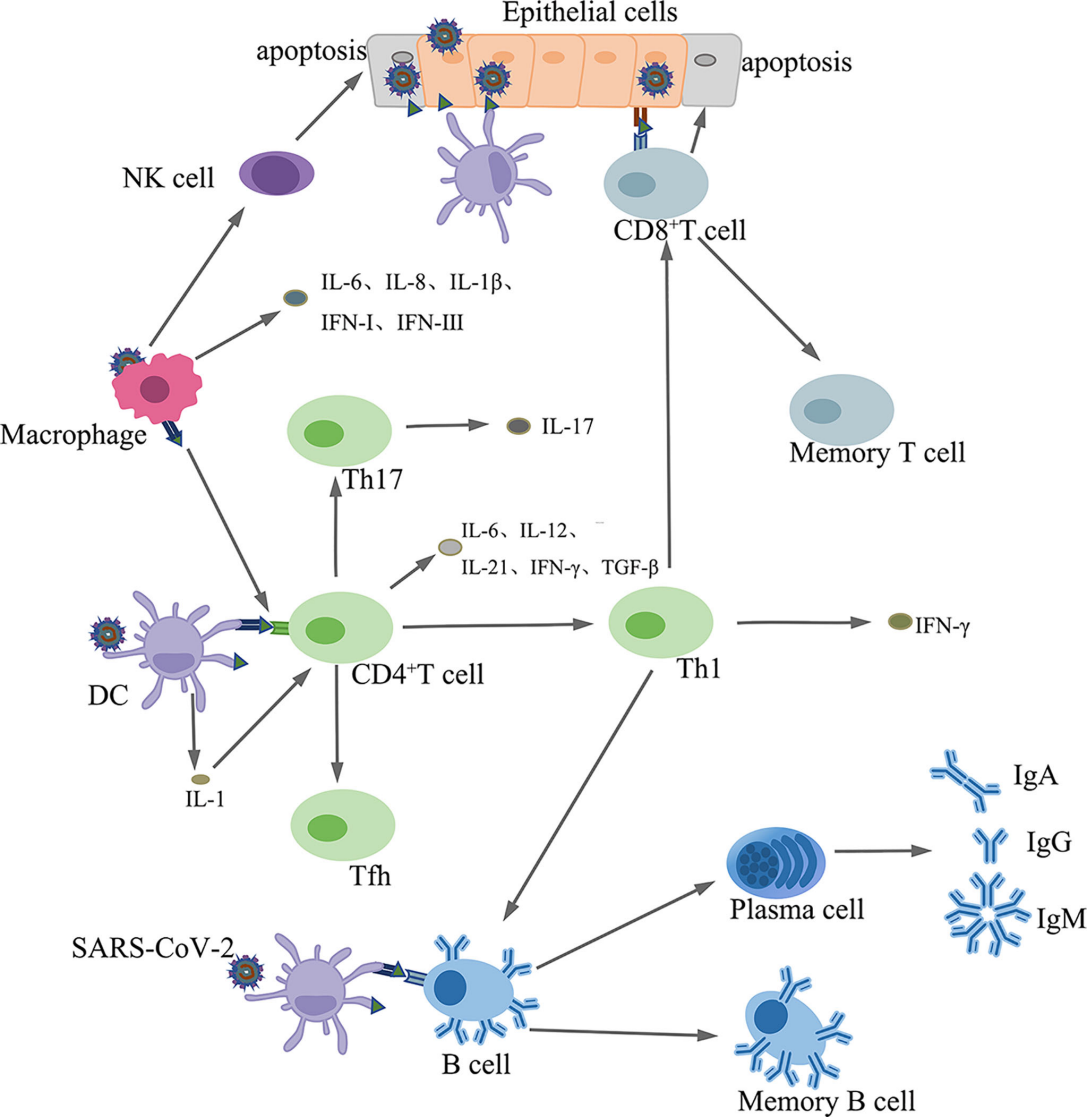
The innate and adaptive immune responses.

APCs present SARS-CoV-2 antigens to CD4^+^ T cells, which differentiate into Th1 sub-types that secret interleukin-12 (IL-12), which further stimulates Th1 cells. Th1 cells also stimulate CD8^+^ T killer cells (Tk) that kill virus infected cells (138). In addition, activated Th1 cells stimulate B cells to produce antigen-specific antibodies (139) [**Figure 4**]. Coronaviruses induce the production of proinflammatory cytokines, such as IL-17, by the helper T cell (Th) 17, which recruits monocytes and neutrophils to the sites of infection. Furthermore, IL-17 promotes the production of inflammatory cytokines, such as TNF-α, IL-1, IL-6, IL-8, and MCP-1 (140, 141).

#### 5.4.2 Humoral Immunity Against SARS-CoV-2

Upon antigenic stimulation, B cells differentiate into plasma and memory B cells. Plasma cells synthesize and secrete antigen-specific antibodies (142) [**Figure 4**].

Neutralizing antibody titers to SARS-CoV-2 peak in the first few weeks after the onset of COVID-19 symptoms and decrease after that at a rate of up to 45% every month (143). In some individuals, SARS-CoV-2 neutralizing antibodies are undetectable within a few months of infection (143), suggesting that serum antibodies do not act as a protective factor for long-term immunity against SARS-CoV-2. A vaccine against the virus aims at increasing the antibody titers to higher levels compared to those induced by natural infection. A vaccine also induces the production of stable memory T and B cells that provide long-term immunity.

Inactivated and live attenuated virus vaccines are whole viruses that induce broader humoral and cellular immune responses (144, 145). However, the mutation of the virus may affect antibody production. The SARS-COV-2 Omicron variant is associated with more efficient cell entry, immune evasion, and increased infectivity (146). Research shows that the third dose of the BNT162b2 vaccine increases the neutralization efficiency of the Omicron variant compared to two doses, but even so, its efficacy is still lower than that against the Delta variant (147). BNT162b2 and mRNA-1273 are less effective in preventing Delta SARS-COV-2 infection but are highly efficacious in severe and critically ill patients (148).

### 5.5 Immune Evasion Induced by SARS-CoV-2

The IFN response is the first line of defense against viruses. However, SARS-CoV-2 strongly suppresses the production of IFN-1 and promotes the production of cytokines (17). SARS-CoV-2 inhibits the production of IFN mainly by (I) evading recognition by the host receptors (149–154) (II), interfering with IFN production (155) (III), blocking signal transmission (54, 156–158), and (IV) inhibiting the function of ISG effectors (58, 159).

Overall, the SARS-CoV-2 proteins mediate immune escape by disrupting the secretion of IFN.

### 5.6 Cytokine Storm

Immune response analysis showed that COVID-19 strongly inhibited the secretion of IFN-1, related to excessive inflammation (160). Clinical studies have shown that the severity of COVID-19 positively correlates with the serum levels of several cytokines, including TNF-α, IL-6, IL-7, IL-17, IL-18, granulocyte colony-stimulating factor (G-CSF), IP10, macrophage colony-stimulating factor (M-CSF), and chemokines. The secretion of cytokines is regulated through the (I) innate immune response signaling pathway (II), angiotensin II/angiotensin type I receptor signaling pathway, and (III) the ACE2 signaling pathway (115, 161).

## 6 Discussion

Some research findings on the oral manifestations of COVID-19 have been reported. The oral manifestations of COVID-19 primarily include gustatory dysfunction and xerostomia, but may also include ulceration, blisters, plaque-like lesions of the oral cavity, herpes simplex, swelling and/or pain in the salivary gland, halitosis, gingivitis, and periodontitis (162, 163). In some patients, xerostomia and gustatory dysfunction are the only manifestations or prodromal symptoms of COVID-19 (35).

The SARS-CoV-2 proteins, especially the S protein, play critical roles in the pathogenicity of the virus. Moreover, mutations might increase the pathogenicity of SARS-CoV-2. SARS-CoV-2 variants are more transmissible, pathogenic, and virulent (164). Indeed, a total of 93-mutations were detected in the SARS-CoV-2 genome. Among them, eight missense mutations occurred in the S surface glycoprotein. Three missense mutations (D^354^, Y^364^, and F^367^) occurred in the RBD of the S protein (165). Mutations may cause conformational changes in the related protein, which changes their antigenic properties (165). Mutations in the RBD domain of the S protein cause the virus to evade neutralizing Abs generated by vaccines (166). Other structural and nonstructural proteins that mediate the pathogenicity of the virus are also targets for COVID-19 treatment and SARS-CoV-2 vaccines’ development.

It has been reported that the healing of oral manifestations of COVID-19 and the regression of SARS-CoV-2 infection occurs simultaneously (162), indicating that the oral lesions might be associated with the infection of SARS-CoV-2. There is evidence that taste changes are caused by SARS-CoV-2 direct infection, which causes cell damage after virus infection, leading to taste dysfunction (122). However, some reports show that oral manifestations of COVID-19 are associated with inflammation, which is associated with immune cell-mediated cell death and tissue damage following SARS-CoV-2 infection (167). The application of omics may help solve this problem. Multi-omics can reveal how COVID-19 interacts with the immune response. The proportion of lymphocytes and neutrophils in the peripheral blood can be used to assess the severity of COVID-19 (168). Decreased lymphocyte counts in patients may lead to insufficient production of immune memory cells, making it difficult to deal with virus re-infection.

The entry of SARS-CoV-2 into host cells is mediated by ACE2 receptors and TMPRSS2. It has been proved that high expression of the ACE2 receptor was found in oral mucosa and salivary glands, and TMPRSS2 was co-expressed with the ACE2 receptor (83, 119, 120), indicating that the oral cavity is susceptible to SARS-CoV-2 infection. These receptors and enzymes facilitate the invasion and the subsequent oral manifestations of COVID-19. Upon entry into the oral host cells, SARS-CoV-2 first initiates a local immune response by inducing the production of IFN. However, SARS-CoV-2 causes a cytokine storm and induces excessive inflammatory responses through immune disorders, which might trigger damage to oral tissues. During the systemic response phase in patients with severe COVID-19, the virus dysregulates the immune response, increases the proportion of neutrophils, and decreases the proportion of lymphocytes. In the end, excessive inflammation damages the involved tissues. Multi-omics studies have confirmed that SARS-CoV-2 affects the immune system and causes immune disorders, suggesting that the pathogenesis of SARS-CoV-2 is related to the innate and adaptive immune responses (169).

## 7 Conclusion

SARS-CoV-2 infects cells of the oral cavity *via* the surface ACE2 receptors and TMPRSS2. The virus binds to its receptors *via* the S protein ligand. Multi-omics analyses further revealed that SARS-CoV-2 dysregulates the immune system mainly by decreasing the expression of IFN-1 and increasing cytokines levels.

## Author Contributions

MH and DW wrote the manuscript. MH, DW, QX, SK, LC, HL, ZY, and WL searched PubMed and Web of Science for citations and prepared figures. All authors contributed to the article and approved the submitted version.

## Funding

This work was supported by the Fundamental Research Funds for the Central Universities (Grant Nos. 2019JCKT-70 and 2020JCXK-45), the Jilin Province Department of Finance (Grant No. JCSZ2019378-8 and jcsz2021893-13), the Jilin Scientific and Technological Development Program (Grant Nos. 20210101010JC and 20200801077GH), and the Changchun Scientific and Technological Development Program (Grant No. 21ZY26).

## Conflict of Interest

The authors declare that the research was conducted in the absence of any commercial or financial relationships that could be construed as a potential conflict of interest.

## Publisher’s Note

All claims expressed in this article are solely those of the authors and do not necessarily represent those of their affiliated organizations, or those of the publisher, the editors and the reviewers. Any product that may be evaluated in this article, or claim that may be made by its manufacturer, is not guaranteed or endorsed by the publisher.
